# Uptake of Contraception Among Adolescent Girls and Young Women PrEP Clients: Leveraging the Opportunity to Strengthen HIV and Sexual and Reproductive Health Integration

**DOI:** 10.3389/frph.2021.684114

**Published:** 2021-07-14

**Authors:** Melanie Pleaner, Zukiswa Fipaza, Khuthala Mabetha, Letitia Greener, Sydney Ncube, Vusile Butler, Mags Beksinska, Saiqa Mullick

**Affiliations:** ^1^Wits Reproductive Health and HIV Institute, University of the Witwatersrand, Johannesburg, South Africa; ^2^MatCH Research Unit, Department of Obstetrics and Gynecology, Faculty of Health Sciences, University of the Witwatersrand, Durban, South Africa

**Keywords:** pre-exposure prophylaxis, contraception, integration, HIV prevention, South Africa, adolescent girls and young women, sexual and reproductive health

## Abstract

The introduction of oral pre-exposure prophylaxis (PrEP) for HIV prevention was a major breakthrough in South Africa (SA). While the initial introduction focused on issues such as the development and implementation of new guidelines, supply, and the development of demand creation strategies, the need to integrate PrEP services with sexual and reproductive health (SRH) services has gained traction both globally and locally. Project PrEP was implemented in eight healthcare facilities and four mobile clinics in three provinces in SA. Using monitoring data from across the four project clusters, and 4,949 clients, over a 21-month period, we conducted an analysis of baseline routine monitoring data to examine contraceptive uptake in adolescent girls and young women (AGYW) initiating PrEP at project sites. Two-thirds of women (62.3%, *n* = 3,083) reported the current use of contraception at baseline, with the most commonly used methods being hormonal injectables (61.9%, *n* = 1,829) and male condoms (19.4%, *n* = 575). A third (32.3%, *n* = 603) of the non-contraceptive users accepted a method at PrEP initiation. From a total of 1,007 (32.7%) current contraceptive users at baseline, 865 (85.9%) chose the same or a different method at this visit. The method uptake at PrEP initiation increased the overall contraceptive prevalence by 12.2 to 74.5%. Data indicated that over a third (38.8%, *n* = 725) who were not using a method at baseline described themselves as consistent condom users. Although a major focus of the project was on PrEP service provision, all women were counseled and offered contraceptive services. The acceptance of a method by a third of non-users was promising; however, more understanding of those who did not take up a method is required. The need to leverage opportunities for the promotion of the integration of HIV and family planning at all levels of PrEP provision was highlighted.

## Introduction

South Africa (SA) has the largest HIV epidemic in the world. In 2019, there were 7.5 million people living with HIV and 200,000 new infections (1). Adolescent girls and young women (AGYW) aged 15–24 are particularly affected, with HIV prevalence among young women nearly four times greater than that of young men (1, 2). Oral pre-exposure prophylaxis (PrEP), comprising the antiretrovirals emtricitabine and tenofovir disoproxil fumarate (TDF), has been a game changer in HIV prevention, with over 90% prevention of HIV when used correctly (3, 4).

Oral pre-exposure prophylaxis was initially introduced into SA *via* several research and demonstration projects, focusing initially on key populations, including men who have sex with men, sex workers, and AGYW (5, 6). The launch of the SA national PrEP guidelines in 2016 (7) was combined with a nationally coordinated training and implementation program, and access to PrEP expanded in a phased approach to include a range of target populations and public sector health facilities, educational institutions, and non-governmental organizations (8). As the availability of PrEP increased, so there was a concomitant interest in exploring different models to improve both access and utilization for AGYW. In addition, there was growing recognition of the need to leverage opportunities to combine HIV services with complementary sexual and reproductive health (SRH) services, with contraception forming an important component of this service delivery package (9–11).

Contraception is identified as a priority strategy in both global and national strategies to improve the health and well-being of AGYW (12, 13). In spite of an enabling contraceptive policy in SA, there are still challenges in contraceptive utilization with young women (14). The 2016 South African Demographic and Health Survey (SADHS) reported that the contraceptive prevalence rate (CPR) in sexually active young women aged 15–19 in the SADHS was 60.4% and slightly higher in females aged 20–24 at 61.3% (15). Despite these rates being one of the highest in Africa, there remains a high rate of adolescent pregnancy and the same survey indicated that 16% of SA adolescent females aged 15–19 have begun childbearing: 1% have given birth, and another 3% were pregnant with their first child at the time of interview (15). Recent data from the national HSRC Youth Survey indicate an unmet need for contraception among young people in SA (14). Among the women who reported being pregnant in the past 5 years, only 10.1% of women aged 15–19 years and 20.9% of those aged 20–24 years desired to get pregnant. Of those who had been pregnant in the past 5 years, only 12.8% in 15- to 19-year olds and 19.7% in 20- to 24-year olds had been using contraception. Although adolescent pregnancy has been a public health challenge in SA for many years, the prevalence of overwhelmingly unintended pregnancies among females aged 15–19 has remained unchanged for the past 20 years (15).

The arguments for integrated SRH and HIV services are supported by the benefits accrued by both clients and healthcare providers in terms of improvements in access, efficient use of resources, comprehensive client-centered quality care, and health outcomes. The interrelationship between HIV and unintended pregnancy particularly underpins this need for integration (16, 17). From a global perspective, this is deemed necessary to attain global development targets, the sustainable development goals (18), as well as the provision of SRH services within a rights-based framework (19, 20).

The literature makes a distinction between linkages and integrated care. Linkages refer to a bidirectional relationship between SRH and HIV embracing policy, programs, services, and advocacy. Integration, a subset of linkages, refers to the combining of several services at the operational or programmatic level, with the provision of combined services occurring at the place, or through a process of structured referrals, usually at the same site (18, 21).

The relative benefits of the bidirectional integration of contraception into HIV services (17, 21–24), and HIV into contraceptive services (18, 23, 25), are well-described and highlight cross-cutting issues such as health system-related issues, improved health outcomes, cost-effectiveness as well as the benefits for the end-user.

Initially, the integration of HIV into family planning services emphasized the promotion of core HIV services such as HIV testing, PMTCT, and antiretroviral treatment (25). More recently, as HIV prevention options have expanded, we have seen the emergence of research and discussion focusing on the potential of the integration of SRH and oral PrEP. An important issue emphasizing the need to include contraceptive services with PrEP provision relates to concern about inconsistent condom use among some PrEP users, and the consequent increased risk of unintended pregnancy (26).

The need to ensure contraceptive services form an intrinsic part of PrEP services for AGYW is particularly important given the range of challenges facing young women with regard to condom use (14), accessing contraception, method utilization, and method continuation (14). The potential of PrEP services being a gateway to access contraceptive services is therefore particularly relevant (5). In addition to the potential benefits of integration, the need to develop the health systems to support integration has also been identified—this includes cross-cutting items across the value chain, including planning and budgeting, supply chain for contraceptive and PrEP commodities, provider training and support, service delivery strategies, end-user support, monitoring and evaluation as well as community engagement and demand creation, framed by youth-friendly service provision (27, 28).

The convergence of the need to improve HIV prevention plus SRH services for AGYW in SA informed the design and implementation of Project PrEP, a comprehensive, real-world HIV prevention program, funded by Unitaid. Initiated in 2018 in SA, the project aimed to improve access, demonstrate service delivery, and provide evidence to guide appropriate and effective models for PrEP delivery framed by comprehensive, quality SRH health services. As a part of this process, we conducted an analysis of baseline routine monitoring data to examine contraceptive use and uptake in AGYW accessing PrEP in the project sites, and how the monitoring and evaluation data can further be improved to capture the bidirectional nature of integration.

## Materials and Methods

### Study Setting and Design

The goal for Project PrEP is to contribute to a decrease in the incidence of HIV in AGYW of age 15–24 years in SA with a primary aim to inform evidence on the appropriate and most effective models for PrEP delivery, in the context of comprehensive integrated SRH and HIV prevention services to AGYW.

Project PrEP is structured around three objectives: (1) to increase accessibility of PrEP (within a comprehensive SRH service package) for the eligible adolescent population in the project implementation areas; (2) to identify and develop effective delivery model and appropriate use of PrEP among adolescents; and (3) to provide evidence (as a country as well regionally and globally) on the use of PrEP in adolescents which is generated and disseminated. The project is working to increase demand for PrEP in high-priority communities where clinics are located by implementing targeted and specially designed demand creation activities. Those interested are linked to care for testing and those negative and at risk offered and provided PrEP as part of a comprehensive SRH package through fixed and mobile facilities.

The service package includes HIV combination prevention options, such as condom promotion, HIV testing, PEP and oral PrEP, and SRH services with a focus on contraception, sexually transmitted infections, and gender-based violence. As PrEP is a relatively new HIV prevention method in SA and is one of the primary objectives for the project, young people may be attracted to the service because of their interest in PrEP. This has the potential of leveraging the opportunity for SRH service integration, such as the promotion of and improved access to contraceptive services. While demand generation activities for the project promoted both HIV prevention and SRH, this was an opportunity to engage on issues such as risk, sexually transmitted infections (STIs), and the prevention of unintended pregnancies in those interested in PrEP.

This implementation science project was undertaken in eight fixed and four mobile healthcare facilities in three provinces in SA (Eastern Cape, KwaZulu-Natal, and Gauteng Provinces). The Eastern Cape included two districts with each district hosting two fixed and one mobile clinic (*n* = 6). KwaZulu-Natal and Gauteng had two fixed and one mobile clinic (*n* = 3, respectively) participating in the project. The study sites were selected to participate in collaboration with the National Department of Health, and in consultation with provincial and district DOH officials. Key factors for facility selection included population density (urban, peri-urban, or rural), client volume at the PHC facility, availability of SRH services within and in the area surrounding the health facility, and geographic size of the catchment area of the health facility. They were selected as facilities in priority areas, which had high HIV prevalence, high rates of teenage pregnancy, and several secondary and tertiary learning institutions in the catchment and so had easy access and the potential to attract young people.

Demand creation activities targeted both communities and health facilities. The youth-friendly services were promoted in the community to encourage AGYW to come to facilities to hear more about what was available. At the facilities and mobile sites involved in the project, health talks and other demand creation activities targeted AGYW. All the staff were trained to provide integrated messages on SRH including PrEP. AGYW who were interested in PrEP or other services were managed according to DoH guidelines (6), and on presentation for a service, the AGYW was asked by the data capturer what services they would like to know more about. The HIV counselor discussed HIV and SRH as part of the risk reduction discussion. The healthcare provider (nurse) discussed risk, PrEP, and HIV prevention, plus assessed which other services are required. Monitoring data were collected *via* a short questionnaire administered by project staff and included demographic characteristics and sexual behaviors, PrEP initiation screening required, HIV prevention service uptake such as HIV testing and counseling, pregnancy testing, and screening services—for example, tuberculosis (TB), gender-based violence (GBV), mental health, and STIs and hepatitis B. This was combined with referrals for any additional services not available on the mobiles. This information was stored in a central health information system for reporting and tracking health service delivery.

Contraceptives available in the fixed sites varied according to commodity supply and staff capacity, but in the main, included injectables [3-monthly intramuscular depot medroxyprogesterone acetate (DMPA-IM), 2-monthly noristerat (norethisterone enanthate/NET-EN], subdermal contraceptive implant, oral contraceptive pills, copper intrauterine device (IUCD), and male and female condoms as per the National Contraceptive Guidelines (29). The mobile sites had injectables, oral contraceptive pills, and male and female condoms; in addition, two of the four mobiles were able to insert implants. Clients were referred to the fixed sites for implant and IUCD insertions. Women were counseled about the different methods during their visit regardless of contraceptive use status and could choose to stay on their existing method or initiate a new method. The non-users of contraception category included a combination of AGYW who had never used contraception and those who had used contraception before but were not currently using a method on the day of their PrEP initiation.

The specific objectives for this analysis were first to examine the level of integration of contraceptive uptake among AGYW users and non-contraceptive users in the sites at first visit. The second objective was to look at patterns of contraceptive uptake, and the third was to reflect on how this data can contribute to improved access and strengthen integration and improve monitoring data to capture integration at service delivery level.

### Study Population and Sample Size

The services in Project PrEP focused on AGYW (<25 years), but women aged 25 years and older and males were able to access the same services. In this analysis, we looked at AGYW (aged 15–24 years), comprising those who were eligible for PrEP services at the 12 project sites between December 2018 and September 2020.

### Data Management and Analysis

The monitoring and evaluation data were collected on hard copy along the client pathway—by the intake data capturer, by the HIV testing counselor, and then the nurse. This is then entered into REDCAP by the data capturer, which is overseen by the data quality monitor. The REDCap (Research Electronic Data Capture) is hosted at the University of the Witwatersrand. REDCap is a secure, web-based software platform designed to support data capture for research studies (30). Data were exported to STATA 15 (StataCorp 2015. Stata Statistical Software: Release 15. College Station, TX: StataCorp LP) for analysis. Data presented are a secondary cross-sectional descriptive analysis of routine monitoring data.

### Ethical Considerations

This project was approved by the University of Witwatersrand Human Research Ethics Committee (M180860), the World Health Organization Ethics Research Committee (ERC) protocol ID: Wits PrEP-AGYW-Main protocol 0003088, and was approved by the Department of Health at the national, provincial, district, municipal, and facility level.

## Results

The overall sample size of AGYW who had visited a facility for any service between December 2018 and September 2020 was 5,376. In this analysis, we looked at those AGYW (aged 15–24), who were HIV-negative, and were offered and accepted oral PrEP at the 12 project sites during this period (*n* = 4, 949). Clients who tested HIV-positive, and who were referred for HIV counseling and antiretroviral treatment, or after HIV testing who did not express an interest in initiating PrEP were excluded (*n* = 427).

Baseline characteristics were collated (Table 1) and showed that two-thirds of women (62.3%, *n* = 3,083) reported the current use of contraception, while a third (27.7%, *n* = 1,866) were non-users. Just under two-thirds (58.7%) were aged 20–24 years, and over two-thirds were learners (still at school) or in post-school education (further education). The proportion in each age category, relationship, and other characteristics were similar across contraceptive users and non-users.

**Table 1:** Baseline characteristics of adolescent girls and young women (AGYW) initiated on pre-exposure prophylaxis (PrEP) (*n* = 4,949).

**Demographic characteristics**	**Contraceptive users at first visit (*n* = 3,083)** ** % *N***	**Non-contraceptive users at first visit (*n* = 1,866)** ** % *N***
**Age**		
15–19	41.3 (1,272)	43.4 (810)
20–24	58.7 (1,811)	56.6 (1,056)
**Relationship status**		
Partner (not cohabiting)	75.4 (2,325)	72.0 (1,343)
Single	17.2 (529)	19.8 (370)
Casual partners	1.8 (57)	3.3 (62)
Open relationship	2.4 (73)	2.5 (47)
Cohabiting	0.6 (20)	1.1 (21)
Married	0.8 (24)	0.3 (6)
Divorced	0.1 (2)	0.1 (1)
Widowed	0.2 (7)	0.1 (1)
Missing	1.5 (46)	0.8 (15)
**Occupational status**		
Learner-primary	0.3 (9)	0.4 (8)
Learner-secondary	32.6 (1,007)	33.3 (622)
Student (post school)^*^	31.6 (975)	36.6 (683)
Student (Other)	5.1 (157)	2.5 (46)
Employed	10.1 (313)	10.2 (191)
Unemployed	18.2 (560)	15.6 (291)
Missing	2.0 (62)	1.3 (25)
Type of facility		
Fixed site	61.3 (1,890)	68.0 (1,269)
Mobile	38.7 (1,193)	32.0 (597)
Population group		
African	99.5 (3,069)	99.4 (1,855)
Other	0.2 (7)	0.3 (5)
Missing	0.2(7)	0.3 (6)
Ever engaged in transactional sex	1.1 (31)	1.3 (24.0)
Self-reported STI in past 6 months	3.3 (101)	2.3 (43)
Always using condoms	42.0 (1,295)	38.8 (725)

* *Technical Vocational Education & Training/University*.

Table 2 shows the different methods that the current contraceptive users were utilizing on the day of their PrEP initiation visit. Injectables were used by two-thirds of AGYW (61.9%, *n* = 1,829) and a fifth (19.4%, *n* = 575) reported male condoms. Similar proportions used oral pills and implants. A small number of contraceptive users (*n* = 6) used contraceptive patches. This method is not available in the public health sector, and these users would have obtained them from the private sector or elsewhere. Condom use consistency was asked in a different section of the questionnaire, and in both user and non-user categories, it was approximately a third in both categories. Among AGYW reporting male condoms for contraception, almost half (47.8%) reported that they were consistent users.

**Table 2:** Contraceptive method type currently used at PrEP initiation.

	**Current users** *n* = 3,083^*^ ** % (*N*)**	Method accepted by non-users^**^n = 1,866 ** % (N)**
Injectables	61.9 (1,829)	42.7 (260)
Male condoms	19.4 (575)	23.3 (142)
Oral contraceptive pills	9.0 (266)	19.7(120)
Implant	8.5 (250)	3.1 (19)
IUCD	0.8 (24)	0.7 (4)
Patch	0.2 (6)	0.0 (0)
Female condoms	< 1.0 (2)	0.0 (0)
No method accepted	N/A	67.6 (1,262)

* *131 AGYW reported to be using a method but method not recorded*.

** *58 New acceptors but method not documented*.

In total, 1,007 (32.7%) of current contraceptive users at PrEP initiation visit chose the same or a different method at this visit. Of these, 865 (85.9%) continued on their current method, while some used the opportunity to change their method.

Table 2 shows the methods accepted by the non-users of contraception at baseline on the same day of PrEP initiation. A third (32.3%, *n* = 603) of the non-contraceptive users accepted a contraceptive method at this visit. The methods chosen by the AGYW were similar in proportion to the current users with the largest proportion (42%) choosing injections. However, although injectables were the most used method, the proportion choosing this method was lower compared to the current users. Oral contraceptive pills accounted for almost a fifth (19.7%) of uptake, 10% higher than the current users. Similarly, 34.6% of those not initiating PrEP accepted a method.

The method uptake at PrEP initiation increased the overall contraceptive prevalence by 12.2 to 74.5%. Two-thirds of the non-contraceptive users (67.6%, *n* = 1,262) did not commence on a method, of which 856 indicated they had no demand, while there were no data available on the remaining 406. The monitoring data indicated that over a third (38.8%, *n* = 725) who were not using a method at baseline described themselves as consistent condom users (used a condom every time they had sex).

In addition to contraceptive services, additional health services were offered to AGYW as part of PrEP initiation and integrated service delivery, including HIV testing and screening for STIs and TB. Although not always documented, 3,947 (79.7%) of AGYW were screened verbally for STI symptoms and 2,460 (50%) were documented to have been screened for TB using a checklist. AGYW were screened for hepatitis B and 185 women positive for hepatitis B were referred for the assessment. A further 15.7% (*n* = 779) were screened for mental health well-being if indicated following a checklist. A total of 73 AGYW received a Pap smear.

## Discussion

The CPR of 62.3% in the group of PrEP initiating AGYW was aligned with that seen in the SADHS 2016 (64% prevalence rate for sexually active, unmarried women) (31). Similarly, the method mix among AGYW aged 15–24 years using contraception mirrored that seen in the SADHS with the injectables being the most common method used at baseline and accepted by the new users, and male condoms the second most utilized method. Although SA has the largest female condom program globally (32), only two AYGW reported being current users and no new user accepted female condoms although they were available at the sites. While it is encouraging to see condoms being reported as a method of contraception, we know that condoms can be less reliable as a method of contraception particularly if used inconsistently and that consistent and correct condom use is a challenge, particularly for AGYW (14, 33). The proportion of AGYW using implants in the AGYW is similar to that reported in the SADHS.

Although a major focus was on PrEP service provision, contraceptive prevalence increased by 12.2–74.5% in the PrEP acceptors. However, all non-users of contraception were offered a method as part of the service package; two-thirds declined. This is potentially a missed opportunity. Unfortunately, the monitoring data did not collect specific information as to the reason for the lack of demand, and reasons for this would require more intensive exploration. The women were, however, given information and could have taken up a method at subsequent visits. Similar numbers of contraceptive users and non-users at baseline reported always using condoms. Condom use was mentioned in both user and non-user contraceptive groups. In the group who did not accept a method at baseline, 38.8% reported always using condoms, indicating that a consistent condom use may not have been perceived as a “contraceptive method” by some AGYW. However, a fifth (19.4%) in the contraceptive user category cited condom use as their method. Condom use continues to be viewed as a STI/HIV prevention strategy rather than a dual method of contraception and STI/HIV prevention (34). This information indicates the need to clarify condom use with AGYW as this method may be under-reported as a contraceptive method. Male and female condoms are available at no cost in all public sector facilities and many non-governmental organizations and community venues. AGYW with access to condoms from non-health facility venues may have not needed to request them from a provider during their consultation, thus reporting no demand. Condoms carried by women remain stigmatized in SA, and AGYW may rely on the provision of condoms by their partners and require partner cooperation for their use (34).

Despite the uptake of hormonal contraceptive methods in the non-users at baseline and the number of consistent condom users, there was still a substantial number of AGYW who did not take up the opportunity to accept an effective method of contraception. Their acceptance of PrEP indicates that they perceived themselves to be at risk of HIV infection and wanted to protect themselves yet may have remained at risk of pregnancy. Despite these youth-friendly services addressing health service-level barriers related to non-use of contraception, there are numerous and multilevel factors at other levels that may be contributing to the lack of demand. At the individual level, factors contributing to risk of pregnancy in AGYW include the lack of knowledge about sex and contraception, young age at first sex, low education, and being out of school (35, 36). Partner-level factors include age disparate relationships (≥5 or more years older) and gender power (in)equity in relationships (37–39). These barriers will need to be addressed to optimize contraceptive acceptability and uptake.

It was encouraging to see that a third of the contraceptive users at baseline chose to use the services available and either continued on the same method or chose a different method on the day they initiated PrEP. We do not know how many of these users had previously used the mobile or fixed service they attended but these data are important as it not only shows that two different services can be accessed in one visit but also that it can be documented. However, challenges arose in collecting follow-up data for each individual as PrEP visits are not necessarily aligned with contraceptive visits, and ensuring that AGYW were tracked across different health facilities where records were not electronic was complex.

Discussing eligibility for PrEP automatically triggers a range of other services that are required in the SA PrEP guidelines, including HIV testing, STI, and hepatitis B and TB screening. This opens the opportunity for discussion of other services, including the need for contraception, TB, and mental health screening. Project PrEP offered the option of PrEP to AGYW regardless of the service they had originally presented for. AGYW who specifically came for PrEP were offered other services. This bidirectional approach ensured that regardless of service, the package was offered.

Although able to provide PrEP, the mobile services are not able to provide the full range of contraceptive methods for several reasons, including the infrastructure to conduct IUCD insertions and only some nurses were trained and able to insert implants. However, they still were able to provide a range of methods and were able to counsel, educate, and refer to fixed facilities. Mobile facilities are important to ensure that the hard-to-reach communities are served with SRH options that are accessible.

Contraception is identified as a priority strategy in both global and national strategies to improve the health and well-being of AGYW (12, 13). This is well-recognized and is reflected as a goal in several regional AGYW programs (DREAMS) and national campaigns (She Conquers) (40, 41), both focusing on contraception and prevention of teenage pregnancy being one of its primary objectives (40). However, there are barriers to young women accessing contraception, and this impacts on effective use and continuation rates on methods (33). Project PrEP sought to improve access to both PrEP and SRH services through integrated service provision, and we looked specifically at how this service provided an opportunity for improved access to contraception for young women.

Emergency contraception is an important intervention to prevent unintended pregnancy for AGYW (42, 43), especially at the community level (44); however, there are no reported monitoring data to report on its use in this project. This needs to be reinforced and brought into the contraceptive method mix as well as capturing this method in routine data collection tools.

The opportunities afforded by strategic and appropriate integration of HIV and contraceptive services are multifold (23): From a service and rights perspective, the integration supports the rights of women to control their own fertility whether HIV-positive or HIV-negative; both provide the opportunity to reinforce discussions about risk, prevention, and options for the combined prevention of HIV prevention and unintended pregnancy, thereby improving health outcomes.

This project focused on the use of PrEP as a potential hook and platform for improving access to contraception. The opportunities presented for integration need to be leveraged and maximized—including finding more effective ways to monitor and measure integration and ensuring that key SRH services such as contraception are combined at all levels of demand creation and that counseling about contraception and contraceptive choices are part of the core PrEP package. Further analysis needs to be undertaken to look at the factors influencing contraceptive use—such as commodity supply and availability, staff training, and harnessing opportunities for integration such as demand creation and risk assessment—and how to ensure these strategies mirror HIV prevention and SRH integration.

## Limitations

There are several limitations to our analysis. The data source was restricted to routine monitoring service data, and there were also changes in the monitoring and evaluation collection tools during the period under discussion, resulting in some data that were missing while more in-depth information such as exact reasons for non-use is not collected routinely. The data do not record the primary service that AGYW came for and other services utilized as a result. Follow-up for PrEP and contraception was challenging for a number of reasons. COVID-19 lockdowns restricted mobile clinics entering communities while repeat contraception, PrEP, and other services were available in the Department of Health Clinic cluster where clients may not have had project data collected.

## Data Availability Statement

The datasets presented in this article are not readily available because: The project is still ongoing but data will be available at a later date on reasonable request to the principal investigators. Requests to access the datasets should be directed to smullick@wrhi.ac.za.

## Ethics Statement

The studies involving human participants were reviewed and approved by University of Witwatersrand Human Research Ethics Committee (M180860), the World Health Organisation Ethics Research Committee (ERC) protocol ID: Wits PrEP-AGYW-Main protocol 0003088 and approved by the Department of Health at national, provincial, district, municipal and facility level. This study used monitoring data and so no parental or guardian consent was required. Written informed consent for participation was not provided by the participants' legal guardians/next of kin because: The data used was from facility monitoring data used by the Department of Health. It was anonymous and not collected for any specific research purpose.

## Author Contributions

SM, MP, LG, ZF, SN, and VB contributed to conception and design of the study. ZF and SN organized the database. KM and MB and MP performed the statistical analysis and data interpretation. MP wrote the first draft of the manuscript. MP, MB, SM, and KM wrote sections of the manuscript. All authors contributed to manuscript revision and read and approved the submitted version.

## Conflict of Interest

The authors declare that the research was conducted in the absence of any commercial or financial relationships that could be construed as a potential conflict of interest.
